# Clinical evaluation of a bone cement-injectable cannulated pedicle screw augmented with polymethylmethacrylate: 128 osteoporotic patients with 42 months of follow-up

**DOI:** 10.6061/clinics/2019/e346

**Published:** 2019-05-28

**Authors:** Zhengdong Wang, Yaoyao Liu, Zhigang Rong, Cheng Wang, Xun Liu, Fei Zhang, Zehua Zhang, Jianzhong Xu, Fei Dai

**Affiliations:** I Department of Orthopedics, National & Regional United Engineering Laboratory of Tissue Engineering, Southwest Hospital, Army Medical University, 400038, Chongqing, China; II Department of Spine Surgery, Daping Hospital, Army Medical University, Daping Hospital, 400410, Chongqing, China; III Department of Orthopaedics, General Hospital of Xin Jiang Military Region, 830000, Xinjiang, China

**Keywords:** Osteoporosis, Pedicle Screw Fixation, Bone Fusion, CICPS, Safety, Effectiveness

## Abstract

**OBJECTIVES:**

To evaluate the safety and efficacy of a novel bone cement-injectable cannulated pedicle screw augmented with polymethylmethacrylate in osteoporotic spinal surgery.

**METHODS:**

This study included 128 patients with osteoporosis (BMD T-score –3.2±1.9; range, –5.4 to –2.5) who underwent spinal decompression and instrumentation with a polymethylmethacrylate-augmented bone cement-injectable cannulated pedicle screw. Postoperative Visual Analogue Scale scores and the Oswestry Disability Index were compared with preoperative values. Postoperative plain radiographs and computed tomography (CT) scans were performed immediately after surgery; at 1, 3, 6, and 12 months; and annually thereafter.

**RESULTS:**

The mean follow-up time was 42.4±13.4 months (range, 23 to 71 months). A total of 418 polymethylmethacrylate-augmented bone cement-injectable cannulated pedicle screws were used. Cement extravasations were detected in 27 bone cement-injectable cannulated pedicle screws (6.46%), mainly in cases of vertebral fracture, without any clinical sequela. The postoperative low back and lower limb Visual Analogue Scale scores were significantly reduced compared with the preoperative scores (<0.01), and similar results were noted for the Oswestry Disability Index score (*p*<0.01). No significant screw migration was noted at the final follow-up relative to immediately after surgery (*p*<0.01). All cases achieved successful bone fusion, and no case required revision. No infection or blood clots occurred after surgery.

**CONCLUSIONS:**

The polymethylmethacrylate-augmented bone cement-injectable cannulated pedicle screw is safe and effective for use in osteoporotic patients who require spinal instrumentation.

## INTRODUCTION

In recent decades, internal fixation with a pedicle screw system has been the gold standard for the treatment of an unstable spine 1 caused by degenerative diseases of the thoracolumbar spine, trauma, or tumors. However, an increasing number of patients worldwide who suffer from osteoporosis have poor bone quality that does not provide sufficient strength for common pedicle screws during internal fixation. Several studies have found that increasing the pullout strength of the pedicle screws can effectively solve this problem. Relevant techniques include increasing the diameter or length of the screw 2; improving the design of the screw-rod 3 or the screw threads 4; choosing a proper insertion angle and trajectory 5; stabilizing the spine with bicortical fixation 4,6; and using expandable pedicle screws 4,7,8 and bone cement-augmented pedicle screws 8-11. However, these strategies have potential shortcomings, such as screw loosening or pullout, screw fracture, vascular or visceral injury, and complications associated with cement leakage 9. Therefore, new techniques that improve the safety and effectiveness of instrumentation are required for the surgical treatment of osteoporosis.

Conventional cannulated pedicle screws augmented with bone cement exhibit several disadvantages. These include unsatisfactory diffusion and distribution of the bone cement 9,12; a fixed screw head design that makes the operation difficult to perform 12; and prolonged operative time and increased blood loss due to the installation and disassembly of the bone cement injecting system. Therefore, we designed a bone cement-injectable cannulated pedicle screw (CICPS) that solved these problems using three radial holes, a flexible screw head, and an injection system.

We previously demonstrated the improved biomechanical stability of the polymethylmethacrylate (PMMA)-augmented CICPS in osteoporotic bone 13,14. In the present retrospective study, we evaluated the long-term safety and effectiveness of the PMMA-augmented CICPS in 128 osteoporotic patients with degenerative spinal diseases.

## MATERIALS AND METHODS

### Patients

The institutional review board of Southwest Hospital, Chongqing, China, approved this retrospective study, and all subjects provided informed consent. The study included a population of 128 patients (29 men and 99 women) with osteoporosis and degenerative spinal diseases who underwent spinal decompression and fixation with PMMA-augmented CICPS at our hospital between March 2011 and March 2015. Diseases included degenerative lumbar spondylolisthesis, lumbar disc herniation with lumbar spinal stenosis, compression fractures, and ankylosing spondylitis (AS).

Osteoporosis in these patients was diagnosed according to the World Health Organization's diagnostic criteria with a T-score≤–2.5 15 by examining the lumbar spine using a Hologic Discovery Delphi SL, QDR^®^ Series (Hologic, Inc., Bedford, MA, USA) with dual-energy X-ray absorptiometry (DXA). Diagnoses of lumbar spondylolisthesis, lumbar disc herniation with lumbar spinal stenosis, and compression fractures were based on clinical symptoms, such as low back pain, radiating pain, numbness, and muscle weakness in the lower limbs and radiological findings in plain-film imaging, computed tomography (CT) 11, and magnetic resonance imaging (MRI). Kyphosis was diagnosed by the appearance of a deformed spine on physical examination and radiologically. Operative management has been advocated for adolescents with progressive kyphosis greater than 70°, patients with progressive kyphosis despite bracing, patients with intractable back pain, and patients with an unacceptable cosmetic deformity 16. All patients had one or more diagnoses; however, we only presented the main diagnosis that provided surgical indications. All patients underwent surgery when their symptoms and signs did not improve after conservative treatment for at least six months. Patients who had blood coagulation disorders or who were allergic to the implants were excluded.

### Implant Design

**Figure 1:**
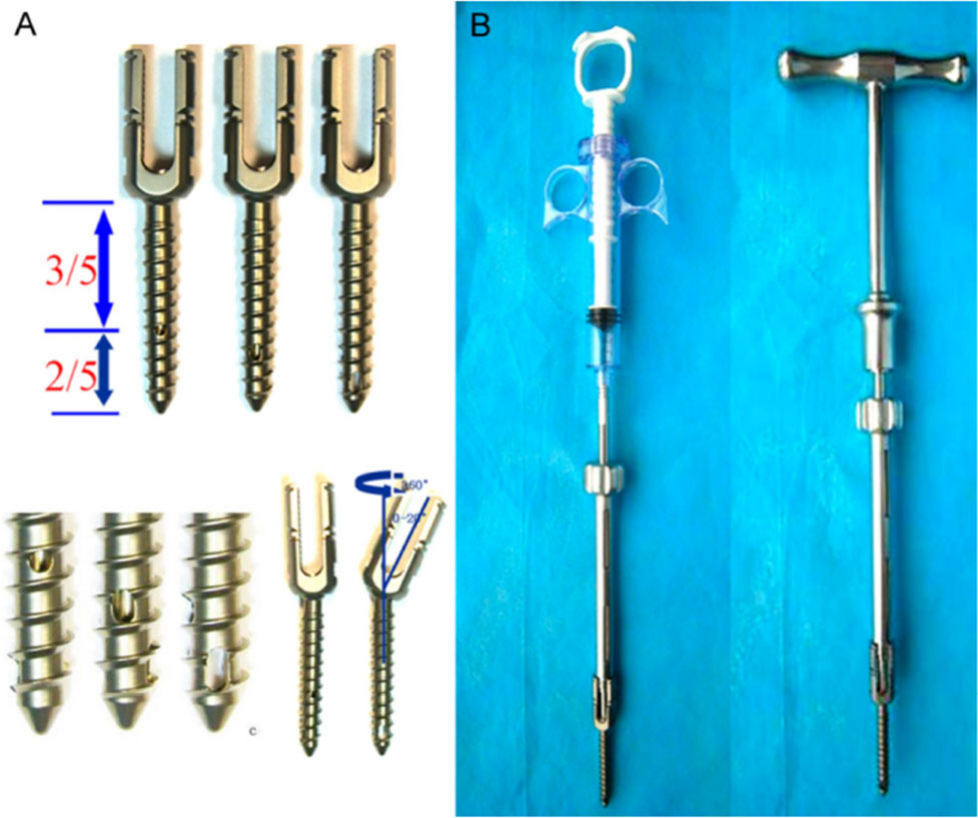
(A) The design of the CICPS; (B) The CICPS connects to the specially designed bone cement syringe and the T-shaped handle through an adapter.

The CICPSs used in this study (Figure 1A) were 3.5-5.0 mm in diameter and 55-70 mm long with a 3-mm pitch (Kanghui Medical Devices, Jiangsu, China). The pedicle screw had a cannulation diameter of 2.2 mm with three radial holes at the distal end (round, 2 mm diameter; oval, 3 mm long and 2 mm wide; and U-shape, 4 mm long and 2 mm wide). The multiaxis or single-axis screw head was designed to facilitate the operative process.

### Surgical Procedures

The patients were positioned prone and were placed under general anesthesia. The surgical procedures included removal of the intervertebral disc, spinal canal decompression, spinal osteotomy and orthopedics, bone graft fusion and posterior internal fixation with PMMA-augmented CICPS, and laminectomy with posterolateral fusion.

The CICPS was implanted with a relatively larger insertion angle to leave more space for the cement. To avoid bone cement leakage to the spinal canal, a screw with a length that was 80-90% of the diameter of the vertebral body was selected. After the CICPS was inserted, PMMA (approximately 1.5 mL) was injected via a dedicated syringe and adapter (Figure 1B and C) and distributed into the surrounding trabeculae bone through the three side holes. An X-ray lateral view of the vertebrate was performed during the PMMA injection to observe the distribution of bone cement. The PMMA injection was stopped when the cement leaked to the posterior part of the screw.

### Postoperative Management

All patients were placed on bedrest for at least 3 days after surgery. Drainage tubes were removed when the volume of drainage was less than 50 mL within 24 hours. Cefazolin sodium (2 g, intravenous drip, every 12 hours) was routinely used for 24 hours to prevent infection. Each patient wore a custom-made lumbosacral or vest orthosis for at least 3 months until fusion was achieved. Patients were encouraged to attempt ambulation 3 days after surgery and allowed to participate in positive activity or go to work depending on his/her recovery and neurological situation.

### Follow-up

All patients underwent follow-up at 1, 3, 6, and 12 months after surgery and annually thereafter. During each follow-up, all patients underwent neurological evaluation and radiological examination. Visual analog scale (VAS) scores were used to evaluate pain severity, and the Oswestry disability index (ODI) score was used to evaluate disability. Any complications, such as cement leakage, infection, and blood clots, were recorded.

All patients underwent pre- and postoperative X-rays and 3D-CT and during each outpatient follow-up. Pedicle screw loosening or pullout was determined using anteroposterior, lateral, and standing flexion-extension lumbosacral plain X-rays according to the distance between the screw tip and the anterior surface of the vertebral body (distance *x*) and the distance from the screw tip to the superior endplate of the vertebral body (distance *y*).

In patients with lumbar spondylolisthesis, the angular displacement and the Taillard index were measured to assess the degree of slipping of the vertebral body 17.

CT scans were performed to determine interbody fusion and cement leakage 6 and 12 months after surgery. Successful fusion was assessed in accordance with Sapkas' and Christiansen's methods 18,19. Screw loosening was considered radiolucent at one millimeter or wider at the bone/screw interface 12. Here, 3D-CT can reflect the shape of the leaky bone cement. For example, when the bone cement seeps into the intervertebral space, it diffuses into the surrounding area and spreads out irregularly. If it flows into a blood vessel, it would exhibit an approximately regular strip shape, which is similar the appearance of the vessel.

### Statistical Analysis

Statistical analyses were performed using SPSS 18.0 for Windows (SPSS, Chicago, IL). The data are presented as the mean ± standard deviation (SD). A paired *t*-test was used to compare the continuous variables at final follow-up to the corresponding preoperative values, including VAS and ODI scores, distances *x* and *y*, angular displacement, and Taillard index. A *p*-value<0.05 was considered statistically significant.

## RESULTS

In total, 128 patients were included in this study (29 men and 99 women; aged 60.7±11.0 y, range, 35 to 83 y; Tables 1 and 2). The cases consisted of main diagnoses of spondylolisthesis, lumbar disc herniation/lumbar spinal stenosis, compression fractures, and AS with kyphosis deformity in 53, 44, 19 and 12 patients, respectively. One or more diagnoses may be noted in one patient. All patients had osteoporosis (bone mineral density T-score≤–2.5).

Additionally, 418 CICPSs were used during the surgeries (Table 1). The mean volume of PMMA injected into each screw was 1.51±0.13 mL (range, 1.2-1.7 mL). The operative time was 216.6±63.4 min (range, 95 to 398 min), and the average blood loss was 553.7±125.9 mL (range, 100 to 1400 mL).

No nerve, blood vessel, or viscera injury occurred during surgery (Table 3). During PMMA injection, no cement leaked into the operative site, and no contamination occurred during the operative procedures. In total, 27 PMMA leakages (6.46%) to the front of 25 vertebral bodies (9.12%) were identified in 20 patients (15.63%) during the procedure. All of these events occurred in prevertebral veins without any observed symptoms. No continuous postoperative bleeding or infection was noted. No symptomatic embolism occurred during hospitalization or follow-up.

**Figure 2:**
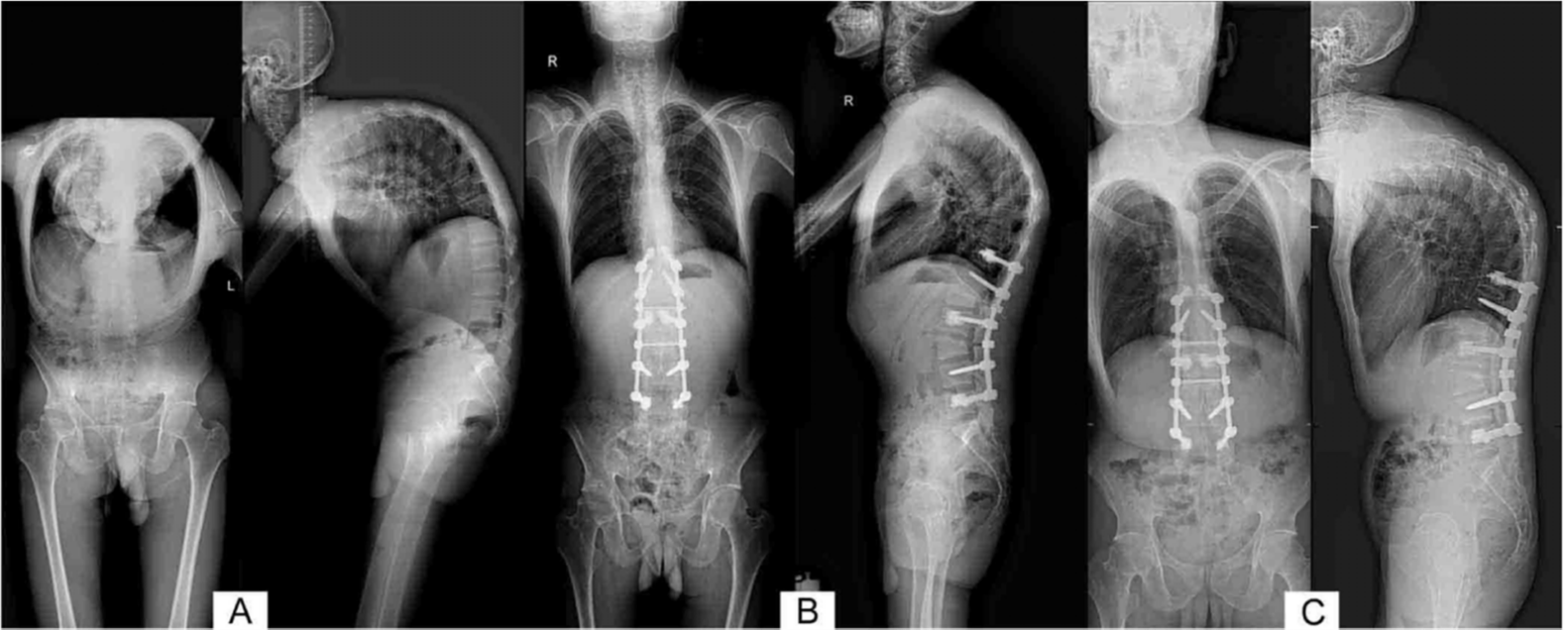
X-ray images. (A) before surgery; (B) immediately after surgery; and (C) at the final follow-up at 52 months in a 31-year-old man with a 4-year history of lower back pain and progressive kyphosis. AS and kyphosis with severe osteoporosis were diagnosed (T-score=–3.0). The patient underwent a partial osteotomy of the key vertebrae without intervertebral fusion. PMMA-augmented CICPSs were used at the ends of the internal fixation instrument, and good spinal correction was obtained.

**Figure 3:**
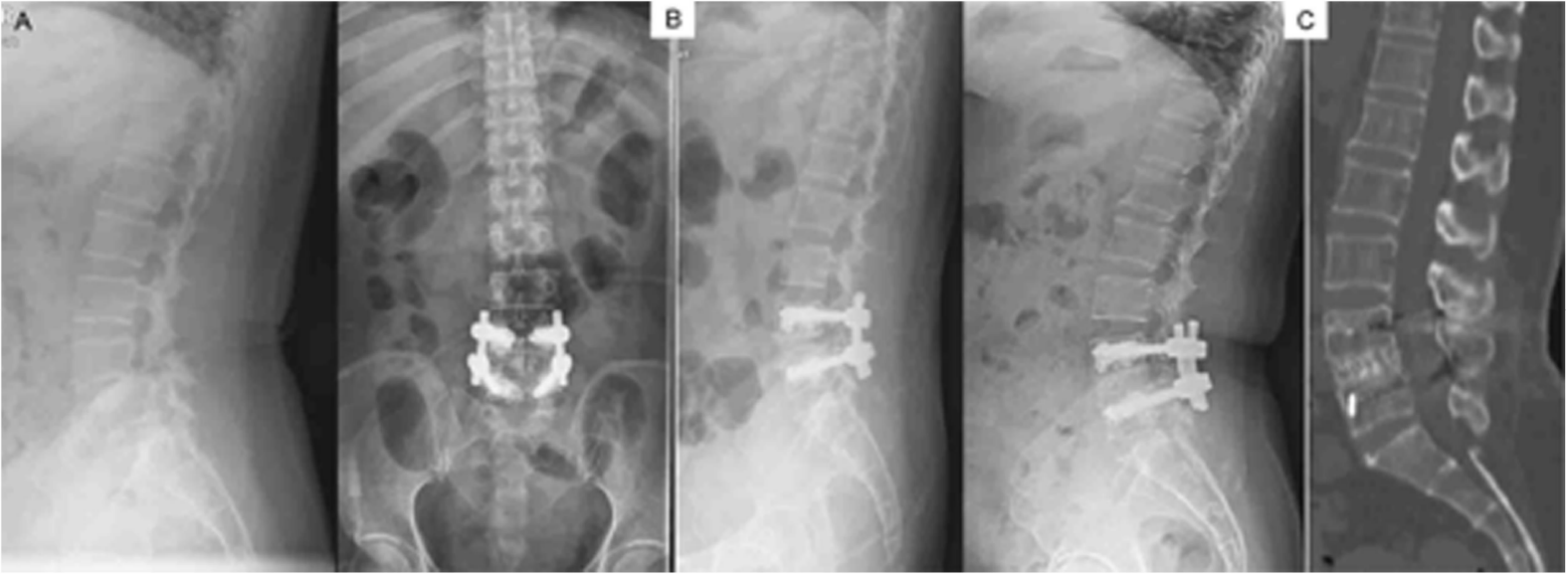
X-ray and CT images. (A) before; (B) immediately after surgery; and (C) at the final follow-up at 57 months in a 67-year-old woman with a 2-year history of lower back pain. L4 spondylolisthesis and severe osteoporosis was diagnosed (T-score=–3.7). The patient underwent transforaminal lumbar interbody fusion with PMMA-augmented CICPSs bilaterally, and the displacement was completely corrected. No screw loosening occurred, and successful fusion was achieved at the final follow-up of 57 months. Low back pain was ameliorated.

All patients underwent follow-up for an average of 42.4±13.4 months (range, 23 to 71 months). Pain and nerve compression symptoms were relieved in all patients postoperatively. The ODI at the final follow-up improved significantly compared with preoperative scores (ODI, *p*<0.001), and similar results were noted in the VAS scores for the low back and lower limbs (each, *p*<0.001, n=87 cases with lower limb pain). Spondylolisthesis and spinal kyphosis deformity were corrected satisfactorily after surgery, and no screw loosening, pullout, or fracture occurred (Table 4, and Figures 2 and 3).

New bone formation within the vertebrae was observed. CT scan results revealed that firm fusions were achieved 6 to 12 months after surgery, and the fusion rate was 100%. No revision was needed during the follow-up. During the follow-up, no patients experienced infection, and pulmonary embolism was not observed.

## DISCUSSION

Although pedicle screws are the workhorse of spinal instrumentation in the adult spine 20, pedicle screw loosening or pullout is the most severe and common problem in osteoporotic patients who undergo spinal surgery that requires spine internal fixation. Although using larger and longer pedicle screws or screws with various designs of screw-rods and screw-threads increase the purchase of the pedicle screws in the inserted vertebrae 5,21-23, bone cement-augmented pedicle screws have been considered more suitable to stabilize and support the degenerating spinal column 24,25. Several experimental and clinical studies have demonstrated that PMMA augmentation can improve resistance to pullout in osteoporotic and normal vertebrae 1,26-28. In the present long-term follow-up study, we tested our redesigned PMMA-augmented CICPSs, which contain three radial holes and flexible screw heads. We found that this new design was safe and effective for osteoporotic patients who needed spinal instrumentation.

Among bone cements, PMMA is the most frequently used in clinical practice due to its low cost, high availability, and strong mechanical properties 8. PMMA is commonly injected directly or via fenestrated screws during vertebroplasty and after the insertion of a balloon in kyphoplasty 8,9,29. The former method is preferable and can result in an approximately 80% increase in pullout strength compared with unaugmented screws 30. The distribution of PMMA injected before insertion of the pedicle screw is not controllable; therefore, there is a high risk of leakage as the screw displaces the cement upon instrumentation 9. PMMA injected via fenestrated screws is better controlled and represents a more effective, safe method.

The presence of side holes in a screw increases the purchase. Chen et al. 31 found that the maximum axial pullout strength of the augmented pedicle screw increased as the number of side holes increased, and bone cement exuded mainly from the proximal screw holes rather than from distal holes. These findings indicate that a cannulated pedicle screw with a side hole closer to the screw head can provide greater maximum axial pullout strength because this screw allows more bone cement to outflow and distribute more widely in less time. However, increasing the number of side holes can cause the pedicle screw to more easily fracture, and a side hole close to the screw head can result in increased risk of bone cement leakage.

To solve these problems, we designed a novel cannulated pedicle screw, the CICPS, which has three side holes of different sizes and shapes. The side holes are arranged from smallest to largest from the distal end of the screw and proceeded along two-fifths of the screw length. The central hollow tract is closed at the screw tip. This design allows almost even pressure in the three side holes during PMMA injection; thus, PMMA can be uniformly distributed around the distal half of the screw. This notion has been confirmed in imaging results 32. Uniform distribution of the cement effectively avoids clinical complications related to cement leakage into the spinal canal. This type of cannulated screw is putatively significantly better compared with other existing types of cannulated screw systems 13,14.

Using less PMMA has some advantages, such as reducing the risk of bone cement leakage and related complications and reducing methyl methacrylate toxicity. In the present study, the mean volume of PMMA used was 1.51±0.13 mL (range, 1.2-1.7 mL), which is considerably less than that reported by (2.89±0.72 mL, range 2.0-5.0 mL) Frankel et al. 33 or (1.83±0.11 mL, range 1.7-2.0 mL) Moon et al. 12. The cement extravasation rate (15.63%) was considerably reduced compared with that in a recent study by Janssen et al. 34 (66.7%), and more than half of the leakage occurred in vertebral fracture cases. The mean BMD (**-**3.53±0.59) was reduced compared with other reports (-2.70±0.20, -2.80±0.42 and -2.90±0.00). Thus, we questioned whether the lower BMD was the true factor leading to the higher extravasation rate. We analyzed the relationship between cement extravasation rate and BMD. The rate was 5.88% (1/17) in cases with a BMD≤-4.0, compared with the rate of 17.31% (9/52) for cases with -4.0<BMD≤-3.0 and the rate of 16.95% (10/59) for cases with -3.0 <BMD≤-2.5, suggesting that CICPS is more suitable in cases with BMD less than 4.0. Low BMD is not the actual cause of the high bone cement leakage rate in vertebral fracture cases, and other reasons, such as the fracture itself, were likely involved. We also noted that the incidence rate of leakage in males (24.14%, 7/29) was higher than that in females (13.13%, 13/99). No obvious differences in age and BMD were noted between males and females.

In the present study, the design of the multiaxis or single-axis screw head and the dedicated syringe and adapter of the CICPS facilitates the surgical procedure and consequently reduces the operative time and intraoperative blood loss. In this study, the mean operative time was 216.6±63.4 min (range, 95 to 398 min), and the average blood loss was 553.7±125.9 mL (range, 100 to 1400 mL).

In the present study, symptom relief and functional improvement was observed in all patients after surgery. This beneficial effect may be associated with solid internal fixation. Pedicle screw loosening and pullout are the main reasons for internal fixation failure with an incidence of 0.6-11% 35. No screw loosening or pullout occurred after a mean follow-up of 42 months. Radiological images during the follow-up revealed no obvious displacement of the pedicle screw tips, and bone fusion was achieved in all patients 6 months after surgery. In addition, several clinical reports have demonstrated that screw breakage results in pedicle screw fixation failure. In the present study, there was no fixation failure and no pedicle screw breakage.

We found that the use of PMMA-augmented CICPS improved the angular displacement and the Taillard index relative to preoperative values in patients with lumbar spondylolisthesis. This finding suggests that PMMA-augmented CICPS is effective for the treatment of lumbar spondylolisthesis with osteoporosis. In patients with AS and kyphosis, although the PMMA-augmented CICPS cannot cure AS or prevent the worsening of deformity at the nonsurgical spinal level, the internal fixation system with PMMA-augmented CICPS benefited the surgical segments.

In summary, we found that use of the novel PMMA-augmented CICPS in osteoporotic patients was associated with good clinical outcomes and the absence of obvious complications during a long-term follow-up of 42 months after spinal surgery. Our study suggests that the internal fixation system with PMMA-augmented CICPS is effective and safe for various unstable osteoporotic spines. According to the results, we also concluded that we should be more cautious in using PMMA-augmented CICPS in cases with a BMD less than -4.0, especially in vertebral fracture patients.

Although we obtained positive outcomes, there were several limitations to this study. First, this study was retrospective in nature and was an open study without a control group. Further clinical randomized controlled studies using this method in a larger number of cases will provide additional data to optimize the procedure, which may contribute to improving healing and maximizing functional outcomes.

## AUTHOR CONTRIBUTIONS

Wang Z drafted the study concepts and designed the study. Liu Y performed the experimental studies, acquired and analyzed the data, and drafted the manuscript. Wang Z and Liu Y contributed equally to this work. Liu Y, Zhang F, Rong Z, Wang C, Liu X and Zhang Z performed the literature research and statistical analyses. Xu J and Dai F are guarantors of the integrity of the entire study and are responsible for experimental guidance and quality supervision. Both Xu J and Dai F participated in coordination and helped drafting the manuscript. All authors read and approved the final version of the manuscript.

## Figures and Tables

**Table 1:** Case report form.

No	Gender	Age	Surgical Indication	BMD,^1^ T-score	Preop^2^ VAS1^4^	Final^3^ VAS1	Preop VAS2^5^	Final VAS2	Preop ODI (%)	Final ODI (%)	Leakage (+/-)^6^	Follow-up (mo)
1	Female	51	Lumbar spondylolisthesis	-2.9	6.00	1.00	5.00	0.00	31.11	2.22	+	69
2	Female	54	Lumbar spondylolisthesis	-3.6	5.00	0.00	0.00	0.00	51.11	0.00	-	67
3	Male	51	Lumbar spondylolisthesis	-2.6	7.00	3.00	5.00	0.00	57.78	6.67	-	66
4	Female	70	Lumbar spondylolisthesis	-5.0	6.00	1.00	0.00	0.00	62.22	0.00	-	66
5	Female	59	Lumbar spondylolisthesis	-3.0	5.00	0.00	0.00	0.00	42.22	0.00	-	65
6	Male	64	Vertebral fracture	-2.9	6.00	1.00	0.00	1.00	60.00	6.67	+	63
7	Female	67	Vertebral fracture	-4.5	7.00	1.00	0.00	0.00	73.33	8.89	-	63
8	Female	66	Vertebral fracture	-2.8	4.00	0.00	0.00	0.00	55.56	0.00	-	63
9	Male	73	LSS	-3.0	1.00	0.00	2.00	0.00	22.22	0.00	-	62
10	Female	58	LDH	-3.1	5.00	0.00	3.00	0.00	46.67	0.00	-	61
11	Male	74	LDH	-3.7	6.00	0.00	3.00	0.00	51.11	0.00	-	61
12	Female	43	Ankylosing spondylitis	-2.8	4.00	2.00	0.00	0.00	50.00	0.00	-	61
13	Female	74	LDH	-2.8	7.00	1.00	2.00	1.00	82.22	11.11	-	61
14	Male	70	Lumbar spondylolisthesis	-3.3	6.00	1.00	6.00	0.00	53.33	0.00	-	60
15	Male	47	Ankylosing spondylitis	-4.5	5.00	0.00	0.00	0.00	53.33	0.00	-	60
16	Male	46	Vertebral fracture	-3.5	6.00	0.00	0.00	0.00	91.11	77.78	+	60
17	Female	60	LDH	-2.6	4.00	1.00	4.00	1.00	40.00	11.11	-	60
18	Female	67	LDH	-4.1	5.00	0.00	5.00	0.00	51.11	0.00	-	60
19	Female	75	Vertebral fracture	-3.5	7.00	1.00	0.00	0.00	60.00	0.00	-	60
20	Female	62	LDH	-2.6	6.00	1.00	5.00	1.00	73.33	15.56	+	60
21	Female	64	LDH	-2.5	5.00	1.00	5.00	1.00	62.22	13.33	+	59
22	Female	63	Lumbar spondylolisthesis	-4.1	3.00	0.00	4.00	0.00	51.11	0.00	-	58
23	Female	64	Lumbar spondylolisthesis	-3.1	5.00	1.00	0.00	0.00	44.44	0.00	-	57
24	Female	59	Vertebral fracture	-3.9	5.00	0.00	0.00	0.00	53.33	0.00	+	57
25	Female	59	LDH	-3.8	4.00	1.00	6.00	0.00	40.00	0.00	-	57
26	Female	61	LDH	-5.2	5.00	1.00	6.00	0.00	40.00	0.00	-	57
27	Female	67	Lumbar spondylolisthesis	-3.3	4.00	0.00	0.00	0.00	31.11	0.00	-	57
28	Female	53	Lumbar spondylolisthesis	-3.7	5.00	2.00	3.00	0.00	42.22	0.00	-	56
29	Female	67	LDH	-5.4	3.00	1.00	3.00	1.00	22.22	13.33	-	55
30	Female	69	LSS	-2.6	6.00	1.00	6.00	0.00	60.00	0.00	-	54
31	Female	59	Lumbar spondylolisthesis	-2.6	3.00	0.00	4.00	0.00	20.00	0.00	-	54
32	Female	46	Lumbar spondylolisthesis	-3.5	3.00	0.00	0.00	0.00	22.22	0.00	-	50
33	Female	57	Lumbar spondylolisthesis	-2.7	2.00	0.00	2.00	0.00	11.11	0.00	-	55
34	Male	60	LDH	-3.1	2.00	0.00	3.00	0.00	8.89	0.00	-	55
35	Female	71	LDH	-2.6	5.00	2.00	5.00	0.00	51.11	20.00	-	54
36	Male	45	Ankylosing spondylitis	-2.6	2.00	2.00	0.00	0.00	62.22	11.11	-	54
37	Female	51	Ankylosing spondylitis	-2.8	2.00	2.00	0.00	0.00	60.00	6.67	-	54
38	Female	59	LDH	-2.6	4.00	1.00	5.00	1.00	40.00	8.89	-	53
39	Female	61	Lumbar spondylolisthesis	-3.5	3.00	0.00	3.00	0.00	31.11	0.00	-	51
40	Female	47	Lumbar spondylolisthesis	-3.2	3.00	0.00	0.00	0.00	48.89	0.00	-	50
41	Female	62	Lumbar spondylolisthesis	-2.9	3.00	0.00	3.00	0.00	31.11	0.00	-	50
42	Female	59	Lumbar spondylolisthesis	-2.7	3.00	0.00	0.00	0.00	40.00	0.00	-	49
43	Female	54	LDH	-4.2	3.00	0.00	0.00	0.00	31.11	0.00	-	49
44	Female	57	LDH	-2.6	5.00	0.00	0.00	0.00	60.00	0.00	-	49
45	Male	69	Ankylosing spondylitis	-4.3	4.00	2.00	0.00	0.00	53.33	22.22	-	49
46	Male	64	Ankylosing spondylitis	-4.2	6.00	2.00	0.00	0.00	82.22	0.00	-	46
47	Female	69	Lumbar spondylolisthesis	-3.0	7.00	2.00	7.00	0.00	80.00	0.00	-	46
48	Male	42	Ankylosing spondylitis	-2.6	4.00	3.00	0.00	0.00	51.11	11.11	-	46
49	Female	69	Lumbar spondylolisthesis	-3.8	5.00	2.00	5.00	0.00	62.22	0.00	-	45
50	Female	64	LDH	-2.6	4.00	0.00	4.00	0.00	46.67	0.00	-	45
51	Female	75	Lumbar spondylolisthesis	-3.6	5.00	1.00	5.00	0.00	57.78	13.33	-	44
52	Female	49	Lumbar spondylolisthesis	-2.7	3.00	0.00	3.00	0.00	42.22	0.00	-	43
53	Female	48	Lumbar spondylolisthesis	-3.0	5.00	1.00	5.00	0.00	31.11	0.00	-	43
54	Male	69	LDH	-2.5	6.00	1.00	5.00	0.00	57.78	8.89	+	42
55	Male	63	Lumbar spondylolisthesis	-2.8	4.00	0.00	3.00	0.00	40.00	0.00	-	42
56	Female	68	Vertebral fracture	-3.5	3.00	1.00	0.00	0.00	40.00	6.67	-	42
57	Female	48	Vertebral fracture	-2.5	3.00	1.00	0.00	0.00	62.22	0.00	+	42
58	Female	63	LDH	-3.2	5.00	1.00	5.00	1.00	62.22	8.89	-	41
59	Female	62	Lumbar spondylolisthesis	-4.5	3.00	0.00	2.00	0.00	31.11	0.00	-	41
60	Female	63	LSS	-2.6	4.00	1.00	2.00	0.00	60.00	0.00	-	41
61	Female	68	LDH	-3.9	4.00	0.00	3.00	1.00	44.44	2.22	-	41
62	Female	57	Vertebral fracture	-3.5	3.00	0.00	0.00	0.00	28.89	6.67	-	41
63	Female	57	Vertebral fracture	-3.3	3.00	0.00	0.00	0.00	44.44	0.00	-	40
64	Female	61	Lumbar spondylolisthesis	-3.4	5.00	1.00	1.00	1.00	60.00	11.11	-	40
65	Female	51	LDH	-2.7	4.00	2.00	5.00	2.00	60.00	0.00	-	39
66	Female	58	Lumbar spondylolisthesis	-3.6	3.00	0.00	0.00	0.00	53.33	0.00	-	39
67	Male	58	LDH	-2.6	3.00	0.00	3.00	0.00	51.11	0.00	-	39
68	Female	65	Vertebral fracture	-3.8	5.00	2.00	4.00	0.00	62.22	17.78	+	36
69	Female	66	LDH	-3.5	6.00	1.00	5.00	1.00	82.22	64.44	+	37
70	Female	72	Lumbar spondylolisthesis	-3.0	4.00	2.00	0.00	0.00	60.00	0.00	-	38
71	Female	66	Vertebral fracture	-3.7	7.00	0.00	0.00	0.00	80.00	0.00	+	38
72	Female	59	LDH	-2.5	4.00	0.00	3.00	0.00	51.11	0.00	-	39
73	Female	57	Lumbar spondylolisthesis	-2.9	4.00	1.00	2.00	0.00	60.00	0.00	-	38
74	Female	54	Lumbar spondylolisthesis	-2.8	6.00	1.00	1.00	0.00	71.11	0.00	-	38
75	Female	68	Vertebral fracture	-4.7	6.00	0.00	5.00	0.00	73.33	0.00	+	37
76	Male	46	LDH	-2.5	5.00	0.00	6.00	0.00	75.56	0.00	-	
77	Male	81	Lumbar spondylolisthesis	-3.6	5.00	1.00	4.00	0.00	51.11	11.11	-	37
78	Female	44	LDH	-2.5	6.00	2.00	4.00	0.00	82.22	0.00	-	36
79	Male	62	Lumbar spondylolisthesis	-2.6	8.00	1.00	1.00	0.00	80.00	22.22	-	36
80	Male	59	Lumbar spondylolisthesis	-2.6	5.00	1.00	1.00	0.00	57.78	0.00	-	36
81	Female	61	Lumbar spondylolisthesis	-2.9	4.00	0.00	2.00	0.00	71.11	0.00	-	36
82	Female	59	LDH	-2.5	5.00	0.00	4.00	0.00	80.00	0.00	-	36
83	Female	59	Lumbar spondylolisthesis	-2.6	4.00	1.00	4.00	1.00	53.33	0.00	-	36
84	Female	48	Ankylosing spondylitis	-2.5	4.00	2.00	0.00	0.00	82.22	0.00	-	36
85	Female	76	LSS	-3.1	4.00	1.00	4.00	1.00	73.33	13.33	-	36
86	Female	76	Lumbar spondylolisthesis	-3.3	5.00	0.00	5.00	0.00	62.22	0.00	-	35
87	Female	66	Lumbar spondylolisthesis	-3.6	4.00	1.00	4.00	1.00	51.11	13.33	-	35
88	Male	35	Ankylosing spondylitis	-3.0	3.00	2.00	0.00	0.00	42.22	0.00	-	34
89	Female	67	LDH	-2.7	3.00	0.00	3.00	0.00	60.00	11.11	-	34
90	Female	64	Lumbar spondylolisthesis	-3.1	4.00	1.00	4.00	1.00	42.22	0.00	-	33
91	Female	56	Lumbar spondylolisthesis	-3.0	3.00	0.00	2.00	0.00	31.11	0.00	-	33
92	Female	63	LDH	-2.8	3.00	0.00	2.00	0.00	40.00	8.89	-	33
93	Female	67	Lumbar spondylolisthesis	-3.7	5.00	1.00	1.00	0.00	51.11	15.56	-	33
94	Female	73	Vertebral fracture	-3.8	5.00	2.00	0.00	0.00	73.33	17.78	+	32
95	Female	57	Vertebral fracture	-3.0	4.00	0.00	1.00	0.00	48.89	0.00	+	30
96	Female	55	Vertebral fracture	-2.5	6.00	0.00	0.00	0.00	71.11	0.00	-	30
97	Female	56	Lumbar spondylolisthesis	-2.5	3.00	0.00	1.00	0.00	40.00	0.00	-	30
98	Female	59	LSS	-2.9	6.00	2.00	4.00	0.00	73.33	13.33	-	28
99	Male	72	LSS	-3.5	3.00	2.00	1.00	0.00	42.22	6.67	-	24
100	Female	60	Lumbar spondylolisthesis	-2.5	6.00	1.00	1.00	0.00	71.11	8.89	-	26
101	Male	50	Vertebral fracture	-3.6	3.00	0.00	3.00	0.00	33.33	0.00	+	27
102	Female	71	LDH	-2.6	3.00	0.00	3.00	0.00	42.22	4.44	-	29
103	Male	59	Ankylosing spondylitis	-2.5	2.00	1.00	0.00	0.00	11.11	0.00	-	28
104	Female	73	LDH	-2.5	5.00	1.00	3.00	1.00	53.33	11.11	-	28
105	Female	52	Ankylosing spondylitis	-2.6	3.00	0.00	0.00	0.00	51.11	0.00	-	27
106	Female	48	Lumbar spondylolisthesis	-2.7	3.00	0.00	0.00	0.00	48.89	0.00	+	26
107	Male	51	Ankylosing spondylitis	-2.9	3.00	1.00	0.00	0.00	42.22	0.00	+	26
108	Male	53	LDH	-3.6	3.00	0.00	1.00	0.00	42.22	0.00	-	26
109	Male	59	Vertebral fracture	-3.4	5.00	0.00	0.00	0.00	62.22	0.00	+	26
110	Female	69	Lumbar spondylolisthesis	-3.0	3.00	1.00	3.00	0.00	51.11	11.11	-	24
111	Female	74	LSS	-4.0	5.00	1.00	4.00	0.00	71.11	0.00	-	26
112	Female	60	Lumbar spondylolisthesis	-4.0	3.00	0.00	1.00	0.00	33.33	0.00	-	26
113	Female	73	LDH	-2.5	3.00	0.00	2.00	0.00	28.89	0.00	-	25
114	Male	71	Lumbar spondylolisthesis	-3.5	3.00	1.00	2.00	0.00	31.11	8.89	-	26
115	Male	64	LDH	-2.9	6.00	0.00	4.00	0.00	82.22	15.56	+	26
116	Female	69	Lumbar spondylolisthesis	-3.8	6.00	2.00	3.00	0.00	84.44	20.00	-	26
117	Female	51	Vertebral fracture	-4.3	3.00	0.00	0.00	0.00	62.22	13.33	-	25
118	Female	62	Lumbar spondylolisthesis	-2.7	3.00	0.00	3.00	1.00	42.22	0.00	-	24
119	Female	61	Lumbar spondylolisthesis	-2.5	2.00	1.00	3.00	0.00	31.11	0.00	+	25
120	Female	64	LDH	-2.7	5.00	1.00	5.00	0.00	62.22	8.89	-	24
121	Female	72	Lumbar spondylolisthesis	-3.6	3.00	0.00	3.00	0.00	37.78	11.11	-	24
122	Male	58	LDH	-2.5	5.00	0.00	4.00	0.00	31.11	0.00	-	24
123	Female	73	Lumbar spondylolisthesis	-2.9	6.00	2.00	4.00	0.00	62.22	2.22	-	23
124	Female	68	LDH	-4.3	6.00	1.00	5.00	1.00	82.22	2.22	-	23
125	Female	49	Lumbar spondylolisthesis	-3.3	6.00	0.00	2.00	0.00	35.56	4.44	-	23
126	Female	83	LSS	-2.7	4.00	0.00	3.00	1.00	51.11	2.22	-	23
127	Female	68	Lumbar spondylolisthesis	-4.7	3.00	1.00	0.00	0.00	42.22	8.89	-	71
128	Female	59	Lumbar spondylolisthesis	-3.0	4.00	1.00	5.00	0.00	51.11	11.11	-	35

1 BMD, bone mineral density.

2 Preop, preoperation.

3 Final, final follow-up.

4 VAS1, VAS low back.

5 VAS2, VAS lower limbs.

6 Leakage did or did not occur.

Abbreviation: LDH/LSS, lumbar disc herniation/lumbar spinal stenosis.

**Table 2:** Baseline demographic and clinical characteristics of 128 patients with PMMA-augmented CICPS in the osteoporotic spine.^a^

		Values
Mean age, y		61.2±11.0 (35 to 83)
Gender M:F, n		29:99
BMD, T-score		-3.20±0.66 (-5.40 to -2.50)
Follow-up, mo		42.4 ± 13.4 (23 to 71)
Surgical indication^b^	Lumbar spondylolisthesis	53 (41.4%)/178
	LDH/LSS	44 (34.4%)/120
	Vertebral fracture	19 (14.8%)/68
	Ankylosing spondylitis	12 (9.4%)/52
	Total	128 (100%)/418
Operative time, min		216.6±63.4 (95 to 398)
Blood loss, mL		553.7±125.9 (100 to 1400)
Bone cement, mL		1.50±0.11 (1.30-1.60)

a Reported as the mean ± SD (range), unless noted otherwise

b n (%)/CICPS, n.

Abbreviations: LDH/LSS, lumbar disc herniation/lumbar spinal stenosis; BMD, bone mineral density.

**Table 3:** Complications in the 128 patients with PMMA-augmented CICPS.

Complication	n (%)						
Cement extravasations*	Surgical Indication	Leakages	Vertebral bodies	Patients, n (%)	Gender M:F, n (%)	Age, y#	BMD, T-score#
	Lumbar spondylolisthesis	3 (1.69%)	3 (2.75%)	3 (5.66%)	0:3 (0.00%:100.00%)	53.33±6.81 (48 to 61)	-2.70±0.20 (-2.90 to -2.50)
	LDH/LSS	7 (5.83%)	6 (6.00%)	5 (11.36%)	2:3 (40.00%:60.00%)	65.00±2.65 (62 to 69)	-2.80±0.42 (-3.50 to -2.50)
	Vertebral fracture	15 (22.06%)	15 (40.54%)	11 (57.89%)	4:7 (36.36%:63.64%)	59.55±8.71 (46 to 73)	-3.53±0.59 (-4.70 to -2.50)
	Ankylosing spondylitis	2 (3.85%)	1 (3.57%)	1 (8.33%)	1:0 (100.00%:0.00%)	51±0.00 (51 to 51)	-2.90±0.00 (-2.90 to -2.90)
	Total	27 (6.46%)	25 (9.12%)	20 (15.63%)	7:13 (35.00%:65.00%)	59.55±8.00 (46 to 73)	-3.19±0.61 (-4.70 to -2.50)
Pulmonary embolism	—	—	—	0 (0.00%)	—	—	—
Infection	—	—	—	0 (0.00%)	—	—	—

* All cement extravasations occurred in paravertebral veins.

# Reported as the mean ± SD (range).

Abbreviations: LDH/LSS, lumbar disc herniation/lumbar spinal stenosis; BMD, bone mineral density.

**Table 4:** Clinical and radiographic data analysis at three time points for the 128 patients with PMMA-augmented CICPSs inserted in the osteoporotic spine; data are reported as the mean ± SD (range).

	Preoperative	Postoperative	Final follow-up
VAS low back, mm	4.34±1.38 (0.00-8.00)	—	0.73±0.78 (0.00-3.00)^a^
VAS lower limbs, mm	3.45±1.49 (1.00-7.00)	—	0.23±0.45 (0.00-2.00)^a^
ODI, %	52.20±17.27 (8.89-91.11)	—	5.19±10.33 (0.00-77.78)^a^
Height, intervertebral space, mm	9.04±3.11 (1.76-16.41)	12.26±1.90 (7.30-17.73)^b^	11.89±1.93 (6.32-16.17)^a^,^c^
Height, vertebral body, mm	16.16±6.23 (5.61-27.73)	22.08±5.01 (13.09-31.37)^b^	22.32±5.21 (12.35-31.57)^a^
Angular displacement, degree	7.09±5.67 (0.34-25.74)	10.75±4.87 (1.37-26.71)^b^	10.16±5.00 (0.87-24.31)^a^
Taillard index, %	27.02±12.14 (9.93-77.85)	6.11±8.30 (0.00-48.02)^b^	6.19±7.74 (0.00-45.27)^a^
*x*, mm	—	4.71±2.94 (0.00-19.21)	4.82±2.98 (0.00-19.74)^c^
*y*, mm	—	8.37±2.96 (0.00-18.85)	8.48±2.91 (0.00-19.01)^c^

a *p*<0.05 Preoperative cf. final follow-up;

b *p*<0.05 Preoperative cf. postoperative;

c *p*<0.05 Postoperative cf. final follow-up.

Abbreviations: *x*, Distance between the screw tip and the anterior surface of the vertebral body; *y*, distance from the screw tip to the superior endplate of the vertebral body.
